# Low-solubility particles and a Trojan-horse type mechanism of toxicity: the case of cobalt oxide on human lung cells

**DOI:** 10.1186/1743-8977-11-14

**Published:** 2014-03-27

**Authors:** Richard Ortega, Carole Bresson, Carine Darolles, Céline Gautier, Stéphane Roudeau, Laura Perrin, Myriam Janin, Magali Floriani, Valérie Aloin, Asuncion Carmona, Véronique Malard

**Affiliations:** 1 Univ. Bordeaux, CENBG, UMR 5797, Gradignan F-33170, France; 2 CNRS, IN2P3, CENBG, UMR 5797, Gradignan F-33170, France; 3 CEA, DEN, DPC, SEARS, Laboratoire de développement Analytique Nucléaire, Isotopique et Elémentaire, Gif-sur-Yvette F-91191, France; 4 CEA, DSV, IBEB, Lab Biochim System Perturb, Bagnols-sur-Cèze F-30207, France; 5 CEA, DEN, DPC, SEARS, Laboratoire d’Analyse en Soutien aux Exploitants, Gif-sur-Yvette F-91191, France; 6 IRSN, Institut de Radioprotection et Sûreté Nucléaire, PRP-ENV/SERIS/LECO, Laboratoire d’Ecotoxicologie des Radionucléides, Saint-Paul-Lez-Durance Cedex 13115, France; 7 Current address: Univ. Nîmes, Lab. Géochimie Isotopique Environnementale, Nîmes F-30035, France

**Keywords:** Toxicity, Lung cells, Cobalt oxide, Particles, Endocytosis, Lysosome, Intracellular solubilization, PIXE, ICP-MS

## Abstract

**Background:**

The mechanisms of toxicity of metal oxide particles towards lung cells are far from being understood. In particular, the relative contribution of intracellular particulate versus solubilized fractions is rarely considered as it is very challenging to assess, especially for low-solubility particles such as cobalt oxide (Co_3_O_4_).

**Methods:**

This study was possible owing to two highly sensitive, independent, analytical techniques, based on single-cell analysis, using ion beam microanalysis, and on bulk analysis of cell lysates, using mass spectrometry.

**Results:**

Our study shows that cobalt oxide particles, of very low solubility in the culture medium, are readily incorporated by BEAS-2B human lung cells through endocytosis via the clathrin-dependent pathway. They are partially solubilized at low pH within lysosomes, leading to cobalt ions release. Solubilized cobalt was detected within the cytoplasm and the nucleus. As expected from these low-solubility particles, the intracellular solubilized cobalt content is small compared with the intracellular particulate cobalt content, in the parts-per-thousand range or below. However, we were able to demonstrate that this minute fraction of intracellular solubilized cobalt is responsible for the overall toxicity.

**Conclusions:**

Cobalt oxide particles are readily internalized by pulmonary cells via the endo-lysosomal pathway and can lead, through a Trojan-horse mechanism, to intracellular release of toxic metal ions over long periods of time, involving specific toxicity.

## Background

Inhalation of cobalt oxide particles can lead to adverse lung effects, mainly as a result of occupational exposure to dust and welding fumes [1-3], including exposure to radioactive cobalt oxide particles in the nuclear industry [4]. Environmental exposure to cobalt oxide particles is associated with airborne particulate matter as a consequence of the combustion of cobalt sources, which generate primarily cobalt oxides [5]. No data are yet available concerning the toxicity of cobalt oxide particles similar to those inhaled in case of accidental occupational contamination in nuclear power plants. However, the increasing industrial use of cobalt nanoparticles has led to many recent *in vitro* toxicological studies [6-13]. The main chemical forms of cobalt micro- and nanoparticles studied are metallic cobalt, cobalt (II) oxide (CoO), and cobalt (II,III) oxide (Co_3_O_4_). These differ greatly in their solubilities, for example more than 50% of metallic cobalt microparticles are solubilized in culture medium after 72 h [6], whereas cobalt oxide microparticles are almost insoluble in water or culture medium [1,14].

The chemical and physical properties of metal particles drastically influence their toxic effects [12,15-17]. Solubilization of the particles, leading to cytotoxic effects related to the free metal ions released and/or the direct toxic effects of metal oxide micro- and nanoparticles through oxidative stress, are among the major mechanisms suggested to be involved at the cellular level. The more-soluble metallic cobalt nanoparticles induce cytotoxicity, ROS formation, and genotoxicity to a greater extent than cobalt ions [6,8,9]. The involvement of dissolution processes in metallic cobalt particle cytotoxicity has been clearly shown for these readily soluble particles [6,8,9,11]. The less-soluble cobalt oxide nanoparticles have been shown to be less toxic than cobalt ions [10], but to cause rapid induction of ROS, with ROS levels higher than those induced by cobalt ions [10,11,13]. Although cobalt oxide particles exhibit a low toxicity *in vitro*, they could lead to long-term adverse effects to lung cells *in vivo*, as they have long retention times in the lung. A small proportion of cobalt oxide particles can remain in the lung for several months or years after inhalation [1,18-20]. For instance, lung retention was 45% of the initial lung deposit six months after human exposure to 0.8 μm Co_3_O_4_ particles [21]. A Trojan-horse type mechanism has been proposed to explain the toxicity of cobalt oxide particles [11], resulting in partial solubilization of particles within cells, especially at low pH within the lysosome, as suggested by *in vitro* studies [14,22]. The major questions that remain to be answered are (i) what amount of cobalt is solubilized in human lung cells, and (ii) is this amount responsible for particle toxicity?

The origin of the toxicity of low-solubility compounds such as cobalt oxide particles is far from being understood and remains very challenging. In toxicological studies, only the extracellular solubilized fraction of the cobalt oxide particles has so far been measured [10,13], showing a very low amount of cobalt released into the culture medium. Although the investigation of particle behavior in culture media is of special relevance for toxicological studies, deeper studies related to the cellular uptake, intracellular solubilization, and behavior of particles are crucial to gain insight into the associated particle toxicity mechanisms.

In this work, we investigated cobalt oxide particle (Co_3_O_4_) toxicity on BEAS-2B human lung cells, and used high-sensitivity analytical techniques that allowed for the first time the discrimination between intracellular solubilized cobalt and non solubilized cobalt in its particulate form. BEAS-2B is a non tumorigenic immortalized cell line that has proven to be a useful model of the airway epithelium for *in vitro* studies of normal lung tissues [23]. A recent study has shown that BEAS-2B cells exhibited the highest homology in gene expression pattern with primary cells and the lowest number of deregulated genes compared with non tumoral lung tissues [24]. Our choice of Co_3_O_4_ particles was motivated by several factors: the good knowledge of the toxicity associated with the soluble cobalt compound (CoCl_2_) in this cellular model [25]; the very low levels of cobalt in cells under physiological conditions, contrary to endogenous metals such as Fe or Zn; the submicrometric, but not nanometric, size of the particles, avoiding the real ‘nano-’ driven toxic effects, although our model is also suited to nanoparticle aggregates; and the known low solubility of Co_3_O_4_ particles, making them a good model for most metal oxide particles. Finally, Co_3_O_4_ particles of this size range are well suited for mimicking radioactive particles encountered in the nuclear industry [19].

We first characterized the size and aggregation of particles and assessed their cytotoxicity on BEAS-2B and also on primary human bronchial (NHBE) cells using ATP-quantification and clonogenic assays. The solubilization ratio of the cobalt particles in the culture and artificial lysosomal fluid (ALF) was assessed. We then followed particle internalization, and identified their internalization pathways. We determined the amount of intracellular solubilized and particulate cobalt fractions upon exposure to various concentrations of cobalt oxide particles, and the *in situ* distribution of solubilized cobalt among the subcellular compartments, such as the cytoplasm and nucleus. Finally, we were able to evaluate for the first time the relative contributions of intracellular solubilized and particulate cobalt fractions on overall cytotoxicity. This methodology can now be applied to the study of other metal particles, especially those containing exogenous elements not present as natural components of the cells.

## Results and discussion

### Cobalt oxide particle characterization

**Figure 1:**
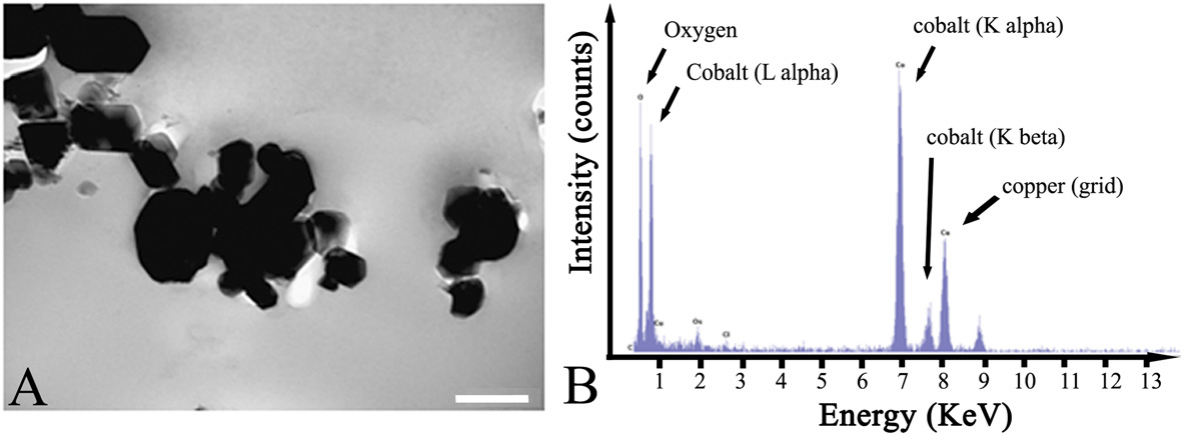
Transmission electron microscopy image of cobalt oxide particles (scale bar: 200 nm) (A) and energy dispersive X-ray spectroscopy microanalysis of cobalt oxide particles (B).

Our purpose was to study the toxicity of particles representative of those involved in the case of accidental inhalation in nuclear power plants. We thus selected the particles according to their estimated average diameter [19]. Physical characterization of these particles had been performed, in part, previously [26]. The morphology of the Co_3_O_4_ particles is shown in Figure 1 panel A, on representative transmission electron microscopy (TEM) images). These images show that the Co_3_O_4_ particles were mainly aggregated, and exhibited a polyhedral structure of heterogeneous size, in the 100 to 400 nm range. Energy dispersive X-ray spectroscopy (EDX) microanalysis of cobalt particles showed that peaks of Co were predominant (Figure 1, Panel B). The presence of Cu was due to the TEM grid used. These results confirmed the high purity of the particles indicated by the supplier (98.4%). These particles are thus suitable for mimicking real cases of contamination, as the particle size in cases of accidental contamination has been estimated to be in the range of 210–850 nm, with a mean value of 410 nm [19]. The distribution of cobalt particles size in LHC9 culture medium suspensions was determined by dynamic light scattering (DLS) measurements. The average size (z-average) and the polydispersity index were determined before and after a 15 min sonication. The data summarized in Table 1 indicate a strong aggregation in the culture medium before sonication. A 15 min sonication was sufficient to disrupt aggregates, the average size of the cobalt oxide particles being 372 nm, with a decreased polydispersity (0.24). In the basal medium (LHCB), which is protein-free, deagglomeration by sonication was not efficient (data not shown) suggesting that the protein corona facilitates deagglomeration [17]. Latex beads (LB-3, 400 nm size), used throughout the study as negative controls, also aggregated in LHC9 medium. Deagglomeration was obtained after a very short sonication (1 min), leading to monodispersed particles of 408 nm (Table 1). These particles, exhibiting a size very similar to that of cobalt particles, were thus a relevant control.

**Table 1:** Average size of cobalt oxide particles and latex beads, before and after sonication in LHC9 medium

	**Before sonication**		**After sonication**	
	**Average size (nm)**	**Polydispersity index (PDI)**	**Average size (nm)**	**Polydispersity index (PDI)**
Co_3_O_4_^*^	1843 ± 279	0.44 ± 0.05	372 ± 101	0.24 ± 0.07
Latex beads (LB-3)**	1024 ± 34	0.26 ± 0.03	408 ± 14	0.06 ± 0.03

(*15 min sonication n = 6, **1 min sonication n = 3).

### Cytotoxicity of cobalt oxide particles *versus* cytotoxicity of cobalt chloride

**Figure 2:**
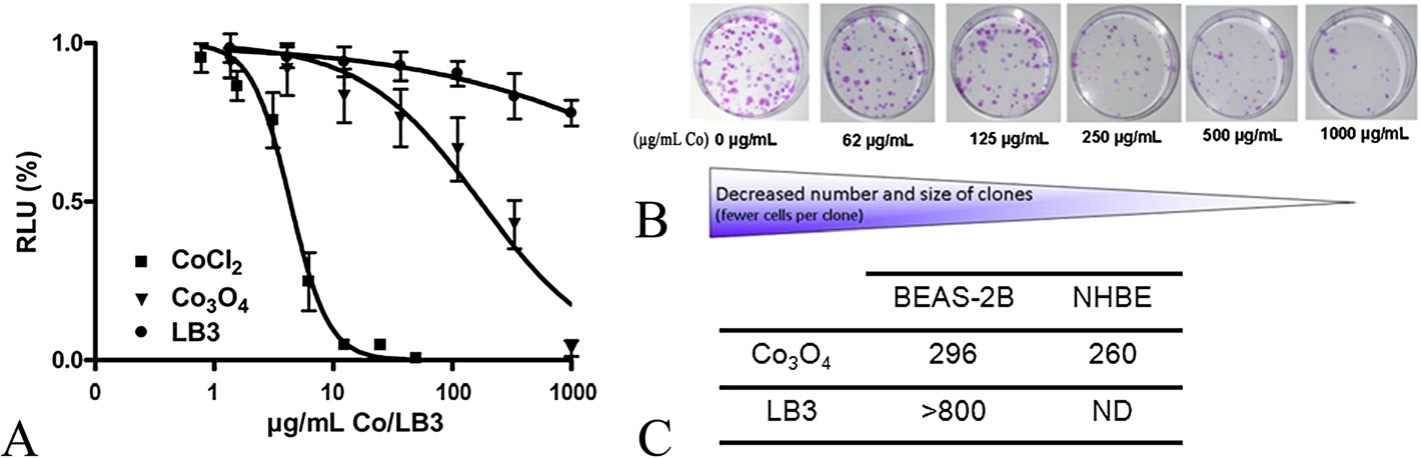
**Toxicity of Co**_**3**_**O**_**4 **_**particles on BEAS-2B and NHBE primary cells.** CellTiter-Glo assay (ATP measurement) **(A)**. BEAS-2B cells were exposed to Co_3_O_4_ particles (0 – 1000 μg.mL^−1^ Co), latex beads LB-3 (0 – 1000 μg.mL^−1^) as a negative control, or soluble cobalt (CoCl_2_) for 72 h (0 – 50 μg.mL^−1^ Co). The cellular ATP content was then evaluated (RLU, Relative Light Unit). Results were obtained from a minimum of three independent experiments, each performed in quadruplicate. Clonogenic assay (proliferation) **(B)**. Example of Co_3_O_4_ particle effects on colony formation of BEAS-2B cells (μg.mL^−1^ Co). Colony numbers were determined following a 24 h exposure to Co_3_O_4_ particles and LB-3 (0 – 1000 μg.mL^−1^ Co and LB-3, respectively) and ten days of culture (time period needed to form colonies). Results were obtained from three to six biological replicates. IC50 (μg.mL^−1^ Co and LB-3, respectively) values obtained after exposure of BEAS-2B and NHBE cells to Co_3_O_4_ particles and latex beads **(C)**.

The cytotoxicity of cobalt oxide particles was evaluated on BEAS-2B cells by measuring the CellTiter-Glo assay after 72 h exposure to increasing concentrations of Co_3_O_4_ particles in LHC9 (0–1000 μg.mL^−1^ Co). Because of the low level of toxicity of cobalt oxide particles [26], we investigated a wide range of concentrations. In parallel, the cytotoxicity of LB-3 latex beads was evaluated for similar concentrations (0–1000 μg.mL^−1^). After 72 h exposure, the sedimentation of 400 nm cobalt oxide particles, as well as latex beads, was fully achieved which validate the cytotoxicity results obtained for these particles suspensions, all the particles being available to the adhering cells. As shown in Figure 2, a dose-dependent decrease in ATP content was observed after cell exposure to Co_3_O_4_ particles. The IC50 (concentration of cobalt for which the ATP content is 50% lower than for non exposed cells) was found to be 170 μg.mL^−1^ Co. The IC25 and IC75 values were, respectively, 50 and 600 μg.mL^−1^ Co. These results show that the toxicity of such cobalt particles is much lower than that induced by soluble cobalt chloride with similar exposure times (72 h), with respective IC25, IC50 and IC75 values of 2.9, 4.4 and 6.5 μg.mL^−1^ Co (Figure 2). These results also demonstrate that at 72 h the toxicity of cobalt chloride is higher than after 24 h exposure [25]. Concerning latex beads, the cell viability decreased very slightly, reaching 75% at the highest concentration, rendering the IC50 determination impossible. This indicates that the toxicity of cobalt particles is not related to their physical properties (size, shape).

We also carried out a clonogenic assay, which has recently been described as a reference method for testing the cytotoxicity of Co_3_O_4_ microparticles [26]. Figure 2B shows the results obtained as colony forming efficiency counts after exposure of BEAS-2B and NHBE cells to various concentrations of Co_3_O_4_ particles (0–1000 μg.mL^−1^ Co). We found that the number of colonies was significantly reduced upon exposure to Co_3_O_4_ particles. We did not formally count the number of cells of each colony but, as highlighted in Figure 2 panel B, we noticed a decrease in their size, thus indicating cytostaticity. After 24 h exposure to Co_3_O_4_ particles, followed by 10 days of culture, the IC50 was estimated to be 296 μg.mL^−1^ Co using this clonogenic assay. These results are consistent with the data obtained using the CellTiter-Glo assay. In addition, these results were validated on NHBE cells (normal bronchial cells), showing that our *in vitro* model represents primary human lung cells quite well in terms of toxicity (Figure 2 panel C). Latex beads were not cytotoxic (IC50 > 800 μg.mL^−1^).

### Cobalt oxide particles solubilization in extracellular medium

Knowledge of the solubilization level of oxide particles able to occur in the extracellular medium, as well as in the intracellular medium, is an essential point in elucidating the mechanisms of toxicity. The potentially released metal ions in the culture medium could be responsible for specific uptake and toxicity mechanisms of soluble ions. For example, it has been observed in human WTHBF-6 bronchial cells that the toxicity of low-solubility lead chromate particles was induced by their extracellular dissolution rather than by their internalization or intracellular dissolution [27].

**Figure 3:**
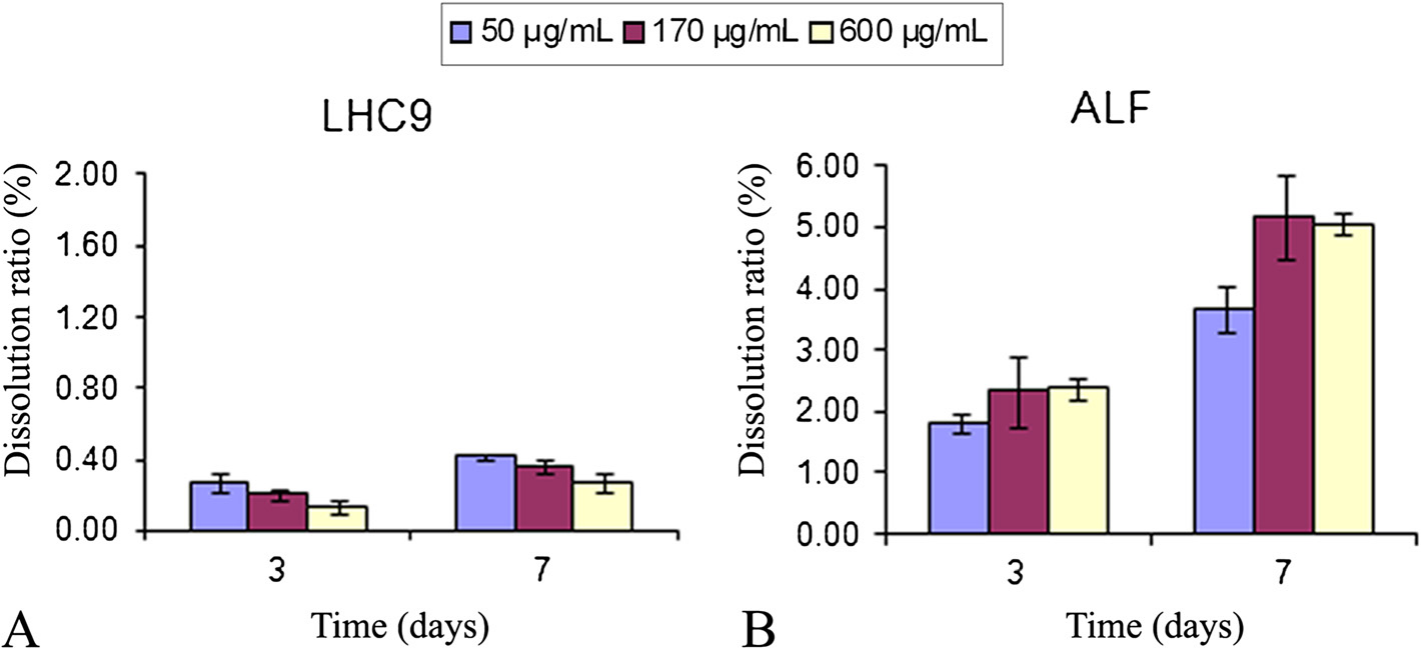
**Cobalt oxide solubilization ratios in LHC9 medium (A) and artificial lysosomal fluid (ALF) (B), for Co**_**3**_**O**_**4 **_**particle suspensions corresponding to IC25, IC50 and IC75 concentrations of cobalt, after 72 h and 7 days.**

The percentage of cobalt solubilization, i. e. the release of ionic cobalt from the particles, in LHC9 culture medium was determined for Co_3_O_4_ particle suspensions corresponding to 50, 170 and 600 μg.mL^−1^ cobalt concentrations (IC25, IC50 and IC75), after incubation periods of three days and seven days (Figure 3 panel A). After incubation, the concentration of solubilized cobalt was measured by ICP-AES/MS (inductively coupled plasma – atomic emission spectroscopy/mass spectrometry). After three days, the dissolution ratios were 0.28%, 0.21% and 0.14% for IC25, IC50 and IC75, respectively. These values were only slightly higher after seven days incubation, ranging from 0.27% to 0.42%. Although the particle dissolution capabilities are influenced by the medium type [28], this result was expected as cobalt oxide particles are only very slightly soluble at neutral pH, such as in LHC9 culture medium. For example, Co_3_O_4_ nanoparticle solubilization ranged from 0.03% to 0.65% after 72 h, for Co_3_O_4_ suspensions corresponding to 88 μg.mL^−1^ cobalt in different culture media and conditions of incubation [10], and was 0.22% after 24 h in DMEM for CoO nanoparticles [12]. In these studies, it was stated that the solubilization is not dependent on particle size. In contrast, cobalt metal nanoparticles/microparticles are more able to dissolve in water and different culture media, at least 44% after 72 h in DMEM [6,8,29] and 40% after 48 h in RPMI medium [30].

In our case, the extracellular cobalt ion content released from Co_3_O_4_ particles in LHC9 medium was too low – i.e. 0.11 μg.mL^−1^ (Table 2) at IC25 (suspensions of Co_3_O_4_ particles corresponding to 50 μg.mL^−1^ Co) – to induce any toxicity on BEAS-2B cells. This concentration is much lower, by almost a factor of 30, than the corresponding concentration of soluble cobalt chloride at IC25 for 72 h exposure, 2.9 μg.mL^−1^ (Figure 2). It can be concluded that the toxicity of cobalt oxide particles on BEAS-2B cells is not due to their extracellular dissolution. Although our study applies to microparticles, this result is in good agreement with what has been suggested regarding Co_3_O_4_ nanoparticles [10,13].

**Table 2:** **Solubilized extracellular and intracellular cobalt content, and intracellular zinc content, measured by ICP-MS, after exposure of BEAS-2B cells to Co**_**3**_**O**_**4 **_**particles for 72 h**

**Co exposure concentration (μg.mL**^**−1**^**)**	**Extracellular solubilized Co (μg.mL**^**−1**^**)**	**Intracellular solubilized Co (fg/cell)**	**Intracellular Zn (fg/cell)**
Control	0	0.4 ± 0.1	57 ± 17
IC25	0.11 ± 0.01	6.5 ± 2.0	103 ± 31
IC50	0.29 ± 0.02	16.5 ± 5.0	135 ± 40

However, cobalt oxide particles are readily solubilized at more acidic pHs such as in ALF medium, an artificial lysosomal fluid mimicking the lysosomal medium, with a pH of 4.5. After their intracellular accumulation, particles are often stored in lysosomal vacuoles where intracellular dissolution can occur, and this involves a higher toxicity than the same concentration of free metal ions, according to the “Trojan-horse” type mechanism [11,31,32]. The impact of pH on the dissolution ratio of cobalt oxide particles in various media has already been investigated. Co_3_O_4_ oxide particles dissolve faster at pH 4.5 than at higher pH [14]. An increase in Co_3_O_4_ particle dissolution has been observed in simple media at pH around 5 [14,22] and reached 10% after 48 h in acetate buffer at pH 4 [22]. In ALF medium, Co_3_O_4_ particles exhibit 1.6% solubility at pH 5.5 after a 24 h incubation period [33]. In our study, the solubilization ratio of Co_3_O_4_ particles in ALF medium was about 2% after three days and up to 5% after seven days (Figure 3 panel B), which is in good agreement with published data. Our results, together with published data, suggest that Co_3_O_4_ particles could enter lung cells and undergo intracellular solubilization in the lysosomal compartment, to exert toxicity through a Trojan-horse-type mechanism.

### Cobalt oxide particle uptake by BEAS-2B cells

**Figure 4:**
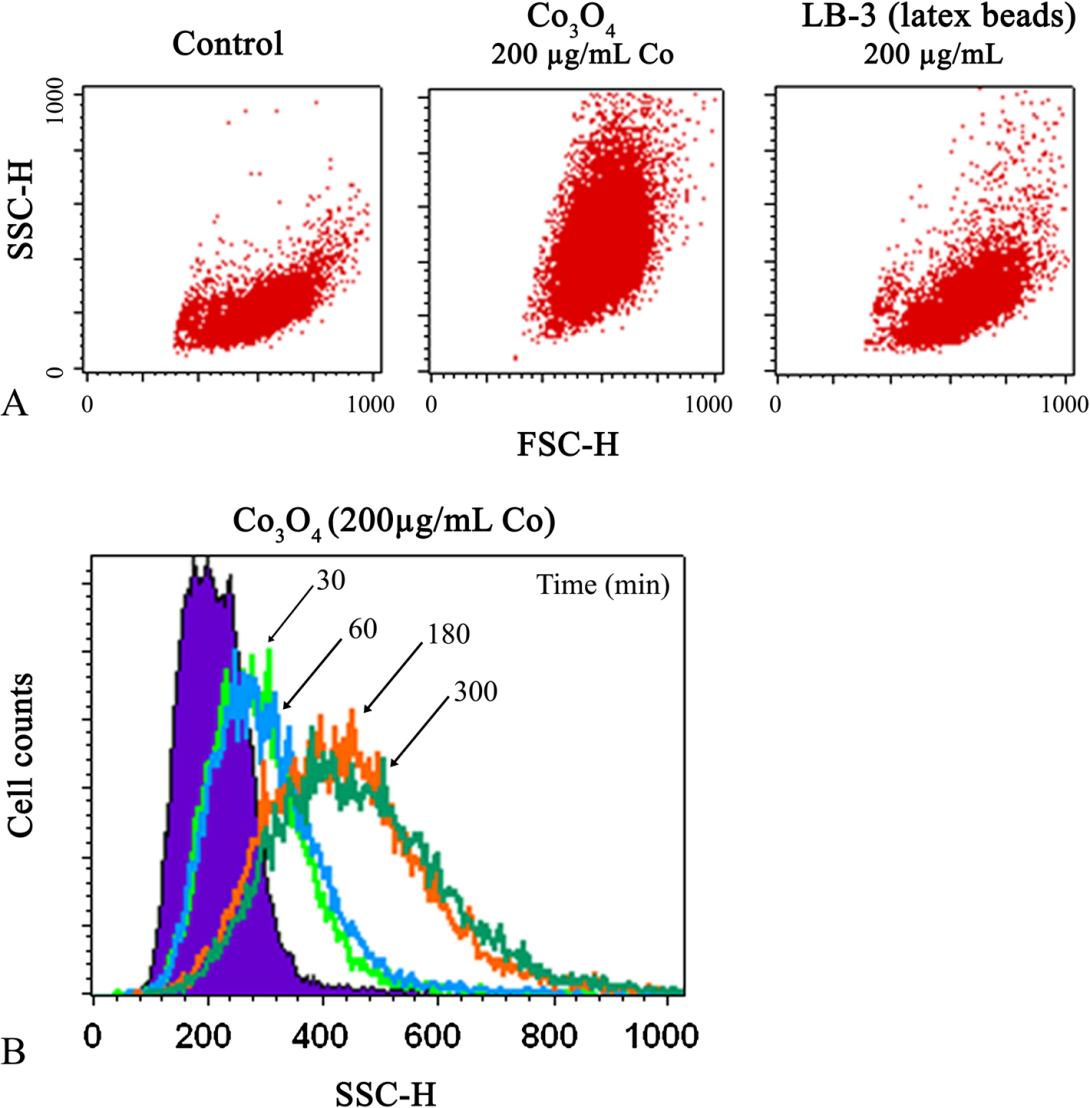
**Flow cytometry analysis of internalization of Co**_**3**_**O**_**4 **_**particles by BEAS-2B cells.** Scatter measurements by flow cytometry after 5 h incubation with cobalt oxide (200 μg.mL^−1^ Co), LB-3 latex beads (200 μg.mL^−1^ LB-3), or without cobalt **(A)**. The results are illustrated by a dot plot representing FSC (forward scatter) versus SSC (side scatter). Flow cytometric histograms of cells exposed to Co_3_O_4_ (200 μg.mL^−1^ Co) for various incubation times **(B)**. 10,000 events were counted for each sample. Data are representative of two independent experiments.

BEAS-2B cells were incubated with Co_3_O_4_ particles (200 μg.mL^−1^ Co) and their uptake was monitored through the increase in SSC (side scatter). As reported in Figure 4 panel A, a significant increase in SSC was observed, and also for LB-3 control latex beads with a size similar to the cobalt particles. The uptake of cobalt oxide particles increased with time (Figure 4, panel B). A noticeable increase in cell granulometry was observed by flow cytometry after only 30 min exposure. This result indicates that cobalt oxide particles were taken up by BEAS-2B cells in a time-dependent manner.

**Figure 5:**
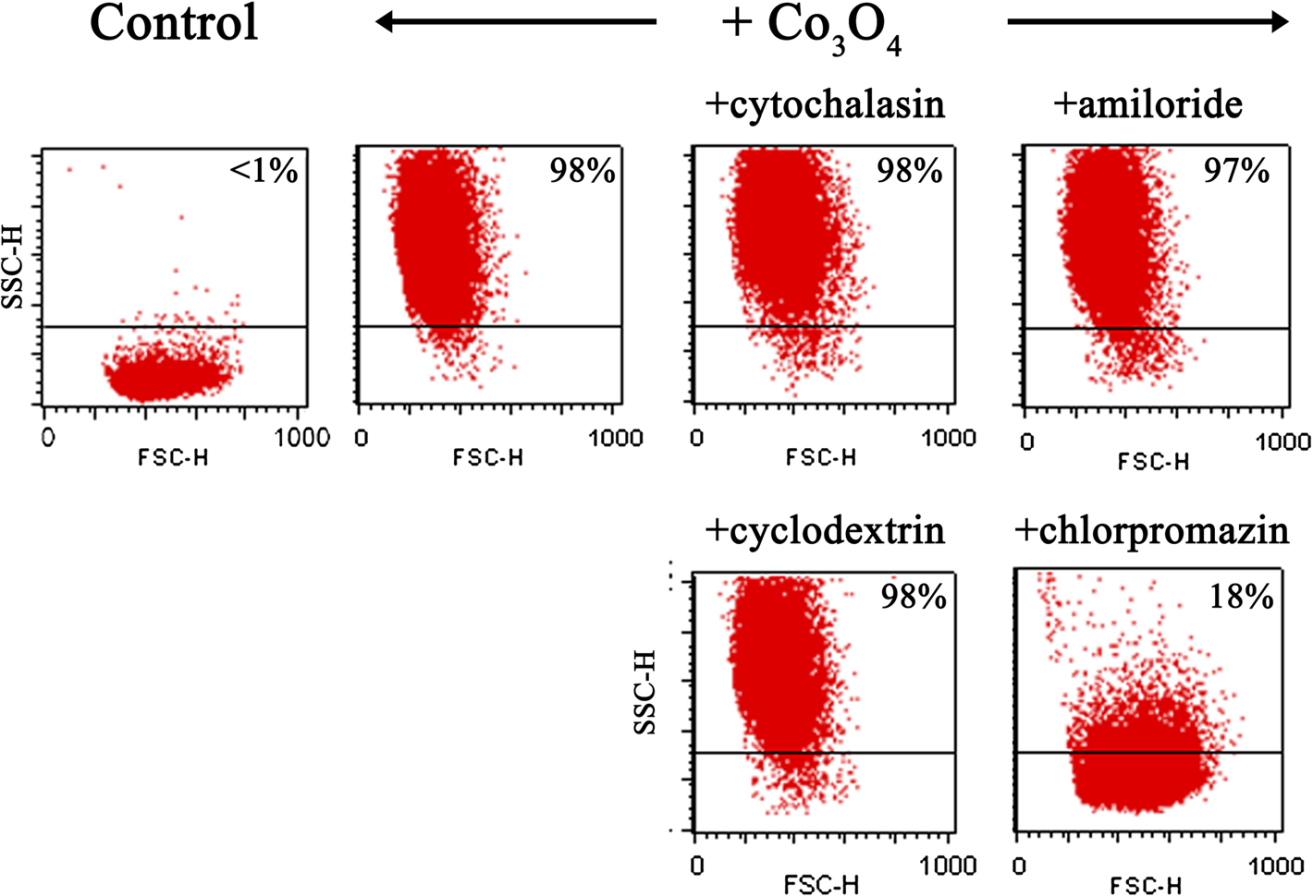
**Flow cytometry analysis of internalization of Co**_**3**_**O**_**4 **_**particles by BEAS-2B cells after treatment with inhibitors.** BEAS-2B cells were incubated with four different inhibitors and cobalt oxide particles (200 μg.mL^−1^ Co). Modulation of SSC is gated, indicated in bold inside each panel. A total of 10,000 events were counted for each sample. Data are representative of two independent experiments.

To better delineate the role of each pathway in the cell internalization of cobalt oxide particles, BEAS-2B cells were treated with known biochemical inhibitors of energy-dependent processes, clathrin-mediated endocytosis, caveolae-mediated endocytosis, and macropinocytosis. We first verified that the chosen inhibitors did not induce any significant cellular toxicity on the BEAS-2B cells under our experimental conditions. The percentage of cells labeled with propidium iodide was less than 10% (data not shown). Figure 5 shows the internalization of Co_3_O_4_ particles in the presence of these inhibitors, as monitored by flow cytometry. Cytochalasin B, which is an inhibitor of actin polymerization, is an inhibitor of macropinocytosis/phagocytosis. In the presence of this drug, the uptake of cobalt oxide particles was unchanged, meaning that phagocytosis or macropinocytosis were not involved in cobalt internalization. Figure 5 shows that amiloride, an Na^+^/H^+^ exchange inhibitor that selectively blocks macropinocytosis [34], also did not inhibit particle uptake. This result confirmed that macropinocytosis is not responsible for cobalt internalization. Methyl-β-cyclodextrin is a cyclic heptasaccharide known to sequester and alter cholesterol-rich domains within the plasma membrane. This drug is an inhibitor of caveolae-mediated endocytosis. In the presence of this drug, similar results were obtained as shown in Figure 5. This indicates that caveolae-mediated endocytosis is not a major internalization pathway for cobalt oxide particles. Internalization of cobalt oxide particles was markedly decreased in the presence of chlorpromazin, a cationic amphipathic drug used to probe clathrin-mediated endocytosis (Figure 5). Because of its amphipathic nature, chlorpromazine can readily be incorporated into the lipid bilayer of the plasma membrane. The resulting increase in lipid fluidity, which in turn inhibits or blocks the formation of membrane invaginations, leads to a strong decrease in cobalt oxide particle internalization. This inhibition was dose dependent (data not shown), and a high inhibition was achieved in the presence of 20 μg.mL^−1^ chlorpromazine (Figure 5). This experiment highlights the role of clathrin-mediated pathways for the internalization of cobalt oxide particles. Different mechanisms responsible for particle uptake are known to be activated, depending on particle size [35]. Xia et al. [36] previously showed on BEAS-2B cells that amino-modified polystyrene nanoparticle (60 nm) toxicity was dependent on caveolar uptake and could be reversed by methyl-β-cyclodextrin. In the present work, we identified clathrin-dependent endocytosis as the entry route for cobalt oxide particle internalization, in agreement with their larger size.

### Intracellular distribution of cobalt oxide particles

**Figure 6:**
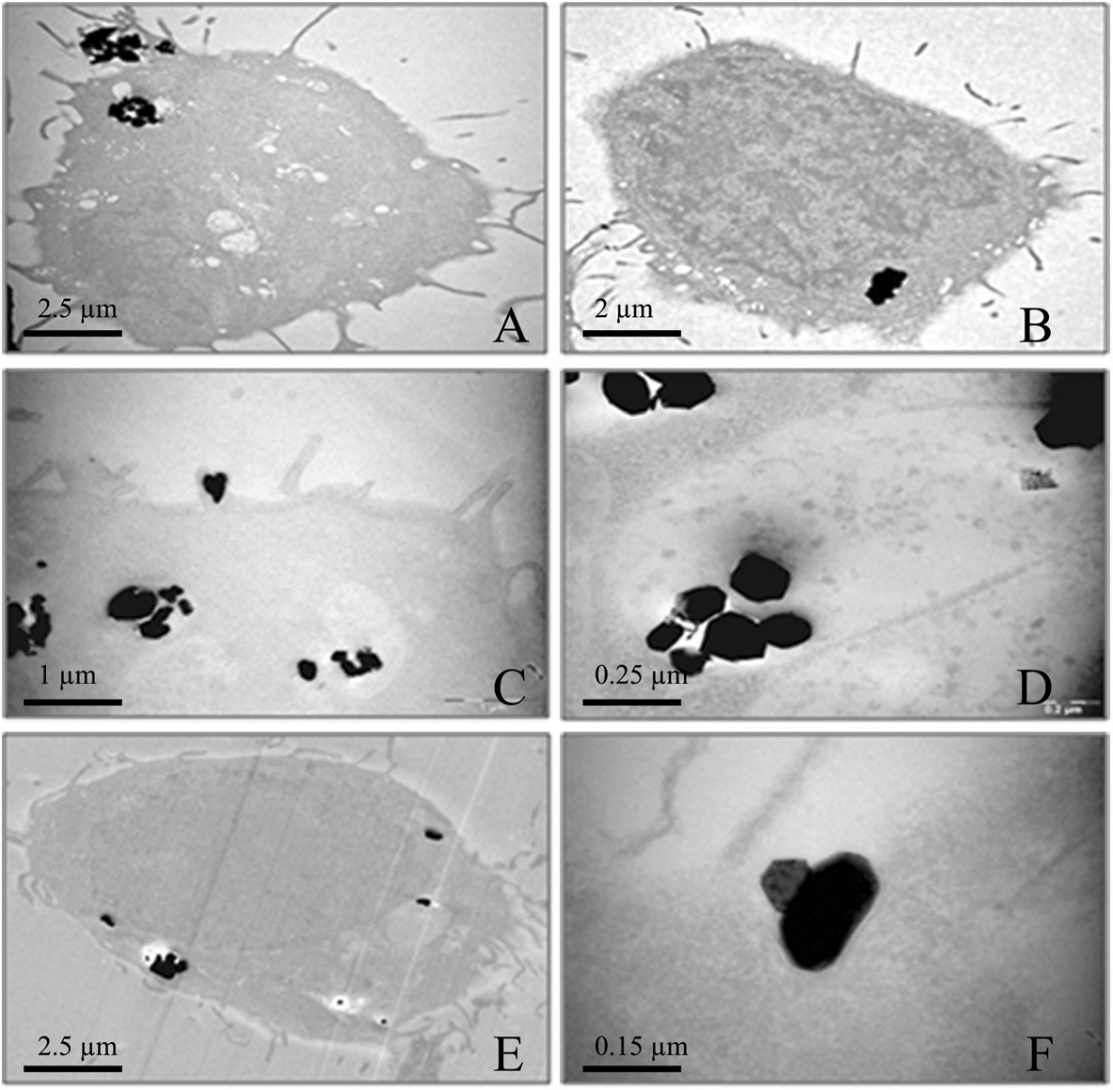
**TEM images of BEAS-2B cells exposed to Co**_**3**_**O**_**4 **_**particles.** BEAS2-B cells were exposed to Co_3_O_4_ particles at 1 μg.mL^−1^ Co **(A, B, F)**, 5 μg.mL^−1^ Co **(C)** or 20 μg.mL^−1^ Co **(D, E)** for 1 h **(A, B)** or 24 h **(C, D, E, F).**

Figure 6,A and B shows two representative TEM images of cells exposed for 1 h to Co_3_O_4_ (1 μg.mL^−1^ Co). Particle internalization as early as 1 h was clearly confirmed by this technique, as also indicated by SSC measurements (Figure 4, panel B). We noted that the quantity of cobalt oxide particles incorporated in the cells was within a wide range, some cells containing a few particles while others could accumulate dozens of them in the same sample. This distribution was also assessed through the wide SSC range given by flow cytometry (Figure 4, panel A). Particles were located in vacuoles which could be lysosomal (Figure 6C and D), and were not observed in the nucleus or dispersed in the cytoplasm. After 24 h, the vacuoles containing particles were localized near the nucleus (Figure 6E). A focus on the cell membrane of cobalt oxide particles being internalized also suggests a clathrin-dependent endocytosis (Figure 6F).

**Figure 7:**
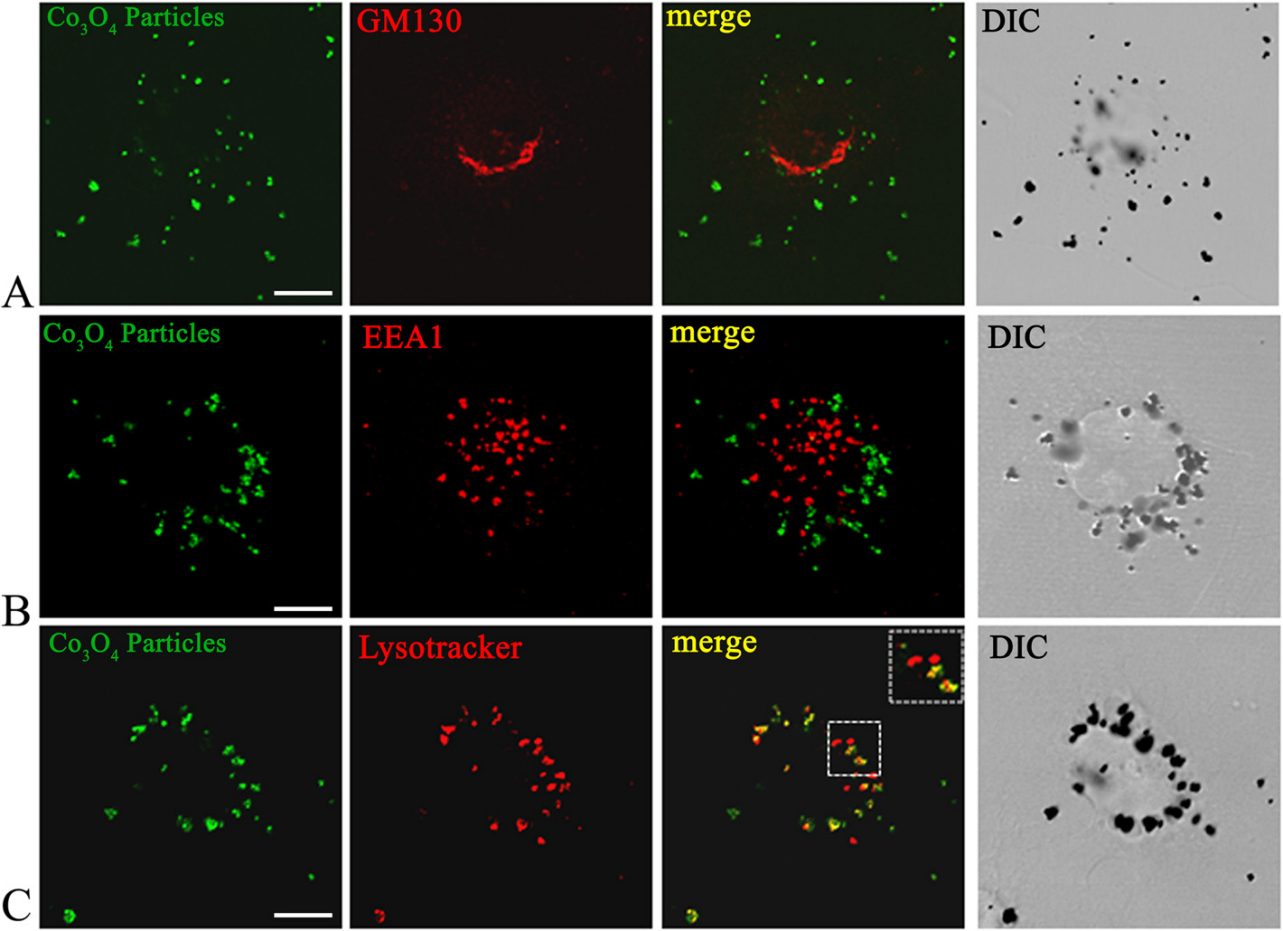
**Confocal microscopy subcellular localization of Co**_**3**_**O**_**4 **_**particles.** Cells were stained for Golgi marker GM130 (red) **(A)**, early endosome marker EEA1 (red) **(B)**, or lysosome marker LysoTracker (red) **(C)**, as described in the materials and methods section, before being analyzed by confocal microscopy. Yellow staining in the merged images indicates co-localization of the cobalt oxide particles (green) with the fluorescent label (red). Scale bar, 10 μm.

In addition to TEM imaging, confocal microscopy was performed on BEAS-2B cells incubated for 24 h with Co_3_O_4_ particles (20 μg mL^−1^ Co) and labeled with organelle markers for the Golgi apparatus (GM130), early endosome (EEA1) and lysosome (LysoTracker Red), as shown in Figure 7. Cobalt oxide particles were clearly seen in reflection mode (green - false color) and DIC (differential interference contrast). Yellow staining in the merged images indicated a co-localization of cobalt oxide particles (green) only with the LysoTraker (red), confirming the lysosomal storage of Co_3_O_4_ particles in human lung BEAS-2B cells (Figure 7 panel C).

The lysosome is the most common intracellular compartment for particles sequestration and degradation. The role of lysosomes in particles intracellular dissolution has been shown in the case of carcinogenic nickel particulate compounds, as recently reviewed [37], zinc oxide nanoparticles [32,38], and iron oxide microparticles [39]. The involvement of lysosomal intracellular dissolution in the release of free metal ions is also suggested in the case of platinum nanoparticles [40]. Our results demonstrate that Co_3_O_4_ particles are stored within the lysosomal compartment and solubilized in ALF, a medium simulating the lysosomal fluid. The lower pH and the action of hydrolytic enzymes within the lysosome may result in enhanced solubilization of cobalt oxide particles into free cobalt ions. Assessment of the intracellular dissolution of cobalt oxide particles was further carried out using two independent and complementary analytical techniques, as described in the next section.

### Intracellular solubilization of cobalt oxide particles

An important key point was to measure the solubilized fraction of cobalt from the Co_3_O_4_ particles inside BEAS-2B cells following exposure of the cells to Co_3_O_4_. This information is crucial to try to determine a relationship between intracellular solubilized cobalt and toxicity. We used two analytical techniques, micro-PIXE (particle-induced X-ray emission) and ICP-MS, which have already shown their complementarity for intracellular cobalt content analysis in BEAS-2B and HaCaT cells [25,41]. PIXE analysis enabled to quantify the intracellular particulate fraction of Co which could not be achieved by ICP-MS. Indeed, during the cell harvesting, involving trypsinization and centrifugation, a fraction of extracellular cobalt oxide particles sediments together with the cells, preventing the accurate separation of intracellular particles from extracellular particles. ICP-MS analyses allowed only the measurement of the solubilized fraction of particles in the cell lysates.

Firstly, elemental analysis of intracellular solubilized cobalt content was performed by ICP-MS after lysis of cells exposed for 72 h in LHC9 to cobalt oxide particles, at IC25 and IC50 concentrations (50 and 170 μg.mL^−1^ of Co), respectively. Table 2 shows that intracellular solubilization of cobalt particles occurred in a dose-dependent manner, leading to 6.5 and 16.5 fg/cell for IC25 and IC50 conditions, respectively. Cobalt oxide particle solubilization in culture medium after 72 h led to 0.11 and 0.29 μg.mL^−1^ released cobalt (Table 2), for Co_3_O_4_ suspensions corresponding to IC25 and IC50. These very low amounts of extracellular solubilized cobalt in the culture medium after 72 h exposure to Co_3_O_4_ particles cannot be responsible for the observed toxicity, as demonstrated by cytotoxicity experiments (Figure 2).

**Figure 8:**
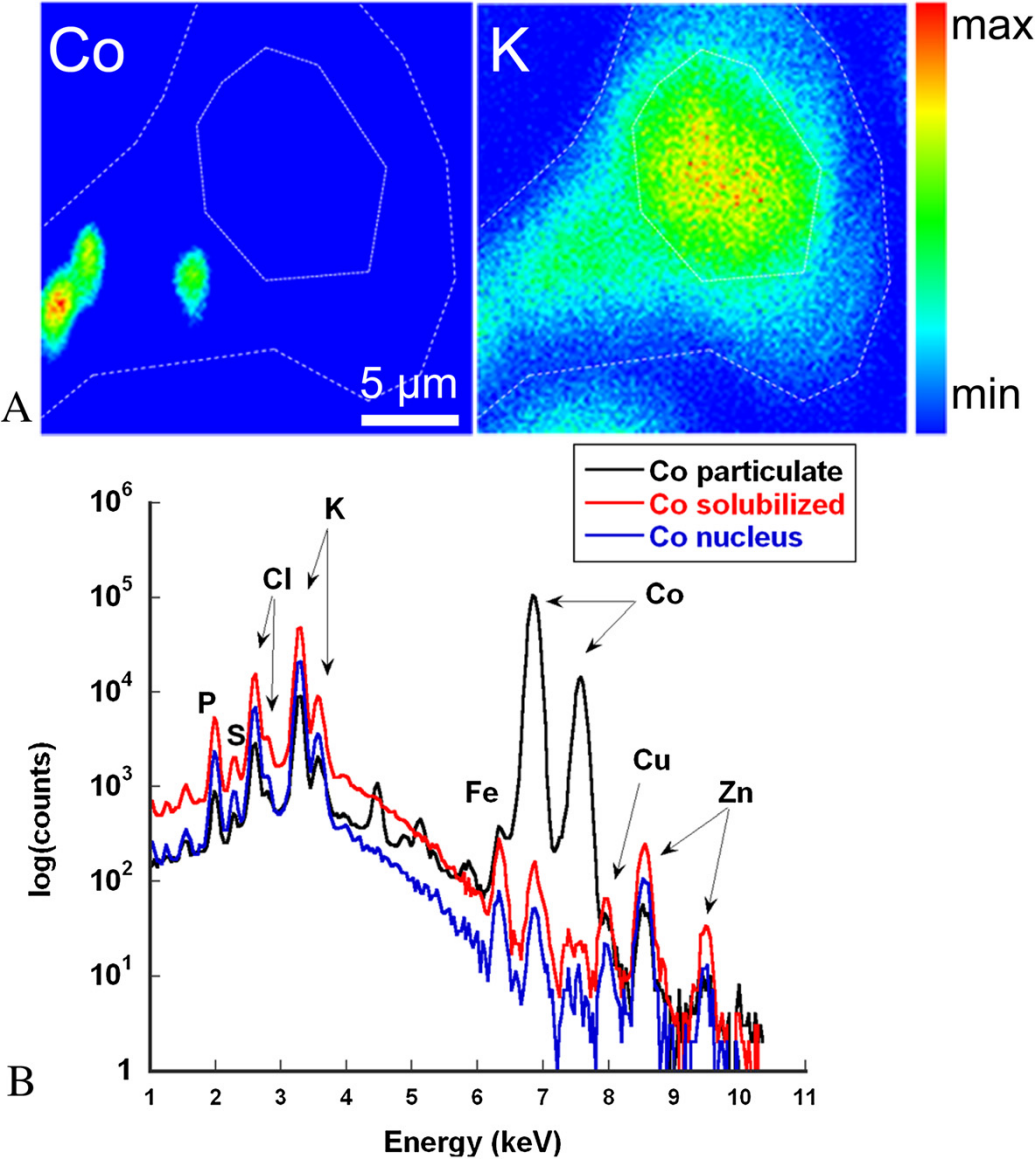
**Example of micro-PIXE analysis of a single BEAS-2B cell exposed to cobalt oxide particles (50 μg.mL**^**−1 **^**Co) for 72 h (IC25).** Potassium distribution delineates the cell boundaries and the nucleus position **(A)**. The highest fraction of cobalt is found within the cytoplasm in its particulate form. A small fraction of cobalt is solubilized as shown by the local PIXE spectrum inside (particulate fraction) and outside (solubilized fraction) cobalt oxide particles **(B)**. In this example, approximately 0.1% cobalt was solubilized and diffused into the rest of the cytoplasm and the nucleus.

To confirm these results, cells were also analyzed by micro-PIXE in order to quantify, *in situ*, the intracellular distribution of the particulate and solubilized fractions of cobalt, as shown in Figure 8, panel A. PIXE analysis is a valuable tool to quantify *in situ* insoluble materials in biological tissues which is usually difficult to perform using analytical techniques that require the prior solubilization of the samples [42]. When performed with a micro-focused beam, PIXE enables to distinguish within cells solid microparticles from the soubilized elemental fraction. Micro-PIXE is better suited to the investigation of microparticles rather than nanoparticles since its spatial resolution is between 200 nm and 800 nm depending on the analytical conditions. PIXE microanalysis offers a better detection sensitivity than TEM-EDX (about a factor of 100), which enables the direct quantification of the slightly solubilized fraction of cobalt in single cells. The chemical map of potassium was used to delineate cell boundaries, as this element is ubiquitously distributed in cells. To determine the intracellular solubilized and particulate cobalt fractions, single cells were imaged and elemental quantification was carried out by PIXE microanalysis in whole cells, either excluding the particles (giving the solubilized fraction) or including only the cobalt oxide particles (giving the particulate fraction), as illustrated in Figure 8, panel B. The mean solubilized intracellular cobalt contents determined by PIXE were 6.7 and 48 fg/cell after exposure to Co_3_O_4_ suspensions corresponding to IC25 and IC50 (Table 3), respectively, confirming the ICP-MS measurements of intracellular solubilized cobalt content after cell lysis.

**Table 3:** **Intracellular cobalt solubilization assessed by single-cell quantitative imaging of cobalt (micro-PIXE) after exposure of BEAS-2B cells to Co**_**3**_**O**_**4 **_**particles for 72 h**

**Co mean ± SD (fg/cell)**	**IC25**	**IC50**
Particulate Co	9500 ± 1650	20,450 ± 23,500
	min 6593 – max 11,483	min 3062 – max 78,486
Solubilized Co	6.7 ± 3.0	48 ± 20
	min 3.4 – max 11.5	min 12.0 - max 70.0
% solubilization	0.07 ± 0.03	0.55 ± 0.50
	min 0.04% - max 0.12%	min 0.06% - max 1.6%

Mean ± SD for the particulate and solubilized intracellular fractions (n = 6 for IC25 and n = 9 for IC50). The solubilization percentage (mean ± SD) was also calculated from each single-cell analysis, and not from the mean solubilized to particulate ratios. Minimal and maximal values are indicated below the mean values.

The PIXE method enabled the determination, for the first time, of the intracellular solubilization ratio of metal oxide particles in single cells, even for a particularly low-solubility compound. Micro-PIXE chemical imaging confirmed that a small fraction of cobalt oxide particles was solubilized in cells. The solubilized cobalt fraction was detected within the cytoplasm and the nucleus of BEAS-2B cells (Figure 8 panel B and Table 4).

**Table 4:** **Summary of the results obtained for the intracellular quantification and distribution of solubilized cobalt after exposure of BEAS-2B cells for 72 h to Co**_**3**_**O**_**4 **_**particles and soluble CoCl**_**2 **_**(both at IC25)**

**Analytical technique**	**Exposure compound**	**Intracellular solubilized Co**
		**Mean ± SD (fg/cell)**
PIXE	Co_3_O_4_	6.7 ± 3.0
		nucleus : 3.3 ± 2.0
		cytoplasm: 3.4 ± 1.5
ICP-MS	Co_3_O_4_	6.5 ± 2.0
PIXE	CoCl_2_	5.4 ± 0.8

The quantitative micro-PIXE results of cobalt content and solubilization ratio in single BEAS-2B cells are presented in Table 3. The solubilization ratio was 0.07% for cells exposed to Co_3_O_4_ suspensions corresponding to 50 μg.mL^−1^ cobalt (IC25), and 0.55% for cells exposed to Co_3_O_4_ suspensions corresponding to 170 μg.mL^−1^ cobalt (IC50). No cobalt was measured in control cells (below the limit of detection of the micro-PIXE method). The solubilization ratios of the particles inside cells are lower than the solubilization ratios in artificial lysosomal fluid (around 2% after 72 h) (Figure 3 panel B), but higher than in culture medium at IC50 (0.21%). A detailed examination of these results strongly suggests a role for lysosomal dissolution of cobalt oxide particles in cells. Endocytosis induces a delay in the lysosomal dissolution process within the cells, as compared with what occurs in a synthetic medium, such as ALF medium. Therefore, the length of intracellular exposure to an acidic pH in the lysosome is less than 72 h. Even if cobalt oxide particle uptake begins early after exposure, the time to particle internalization can greatly vary from one cell to another, and can be as different as a few minutes to almost 72 h. This is particularly emphasized for the IC50 condition, with a wide range of variability in the intracellular solubilization ratio, from 0.06% to 1.6%. In this exposure condition, the extracellular cobalt solubilization ratio (0.21%) is lower than the intracellular solubilization ratio (0.55%), and in some cases this latter is as high as 1.6%, which can be explained by lysosomal dissolution at acidic pH.

The intracellular solubilization of cobalt oxide particles was confirmed for the first time using two independent analytical approaches. Only a very small amount of cobalt was solubilized in BEAS-2B cells after 72 h exposure to cobalt oxide particles, which is in agreement with the known low solubility of this compound. The mean solubilized intracellular cobalt content determined by PIXE was 6.7 fg/cell after exposure to Co_3_O_4_ suspensions corresponding to IC25, confirming the elemental ICP-MS measurements of intracellular solubilized cobalt content after lysis of cells, which was 6.5 fg/cell (Table 4). The quantitative analysis of solubilized cobalt content in the nucleus and the cytoplasm of BEAS-2B cells suggests a homogeneous distribution, in good agreement with our previous results obtained with cells exposed to soluble cobalt (CoCl_2_) [25]. At IC50, PIXE and ICP-MS data are less in agreement with respective values of intracellular solubilized Co of 48 and 16.5 fg/cell. Micro-PIXE measurements could be slightly overestimated due to the contribution of cobalt oxide particles especially as the number of particles incorporated within BEAS-2B cells is very high at IC50.

Another piece of evidence for cobalt oxide particle intracellular dissolution can be inferred from the study of cobalt-induced hypoxia due to the cellular effects of cobalt ions in solution. Cobalt ions are known as “hypoxia-simulating” agents [43]. We were able to observe a strong stabilization of the hypoxia-inducible factor 1 (HIF-1) by both cobalt forms, soluble cobalt chloride and particulate cobalt oxide (data not shown). A hypoxia-like response induced by the particulate cobalt form could result from its partial solubilization in late endocytotic vesicles, a mechanism which has been described in macrophages from rabbits exposed to cobalt oxide particles of a similar size as those we studied [22]. As hypoxia seems to be an important mechanism in cobalt-induced lung inflammation [44], the fact that cobalt oxide can stabilize HIF-1, even if indirectly, is a key point when considering the mechanisms of toxicity of these particles.

### Impact of intracellular solubilization of cobalt oxide particles on toxicity

The next major question is to determine if the toxicity induced by the particles is associated with the particles *per se*, with the intracellular solubilized cobalt fraction from the particles, or a combination of both. During the solubilization of cobalt oxide particles, Co(II,III) particles are reduced into solubilized Co(II), a reaction that can produce free radicals in cells. On the other hand, cobalt toxicity associated with soluble compounds has been documented by *in vitro* studies, mainly performed using cobalt chloride, CoCl_2_. These studies have shown that cobalt is genotoxic [45,46], induces oxidative stress [47], apoptosis [48], and is a hypoxia-simulating agent [49]. Micro-PIXE analysis was thus performed on BEAS-2B cells exposed to soluble cobalt chloride in similar toxicity conditions as those used for Co_3_O_4_ particles (IC25 and IC50 of Co, for 72 h). It should be noted that, conversely to Co_3_O_4_ particles, cobalt chloride is present totally as soluble species in LHC9 medium [25]. Intracellular cobalt contents of, respectively, 5.4 and 9.6 fg/cell were found after exposure to CoCl_2_ at the IC25 and IC50 for 72 h (2.9 and 4.4 μg.mL^−1^, respectively). It can be noted that the global total intracellular cobalt content is much higher after exposure of the cells to Co_3_O_4_ particles than to soluble CoCl_2_ (respectively, 9500 and 5.4 fg/cell at the IC25 of Co). This indicates that endocytosis is a very efficient uptake pathway in comparison with specific transport or ionic pumps that can be involved in soluble cobalt incorporation. The intracellular solubilized cobalt content after exposure to soluble CoCl_2_ was very close to those measured after exposure to cobalt oxide particles (Table 4). In addition, an increase in intracellular zinc content was measured by ICP-MS after lysis of the BEAS-2B cells exposed to Co_3_O_4_ particles for 72 h to the IC25 and IC50 of cobalt, from 57 fg/cell in the control cells, to 103 and 135 fg/cell, respectively (Table 2). In our previous work [25], a modulation of zinc homeostasis has been observed upon BEAS-2B exposure to the soluble cobalt compound, CoCl_2_. It is noticeable that exposure of BEAS-2B cells to soluble cobalt and to particulate cobalt oxide induced a similar increase in intracellular zinc, reflecting a modulation of zinc homeostasis.

**Figure 9:**
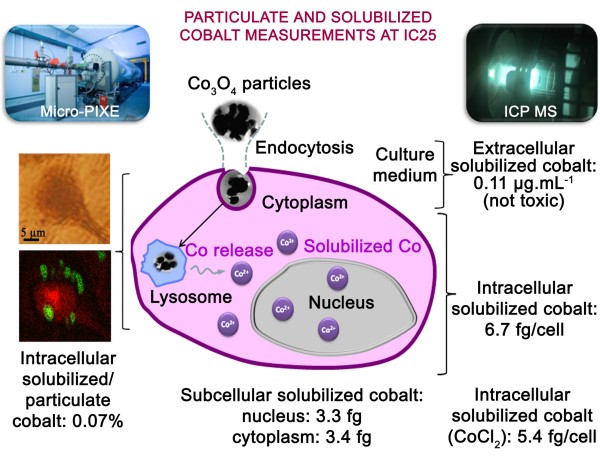
Schematic overview of the experimental design and of the main results for particulate and solubilized cobalt quantitative analysis at IC25.

These results indicate two important mechanisms of toxicity: 1) the cobalt oxide particles are readily internalized by endocytosis via the clathrin-dependent pathway, then 2) they are partially solubilized within BEAS-2B cells and, even if the intracellular solubilized cobalt fraction is very low compared with the total cobalt content, this solubilized fraction is responsible for the overall toxicity. This latter aspect could be demonstrated as the intracellular solubilized cobalt content after exposure to Co_3_O_4_ particles (6.5 fg/cell) is similar to that after exposure to the soluble compound, CoCl_2_ (5.4 fg/cell), for the same conditions of cytotoxicity (IC25). Such mechanisms have never been demonstrated for low-solubility particles such as cobalt oxide particles. Our results also explain the differences in cytotoxicity of more-soluble cobalt metal particles and poorly soluble cobalt oxide particles. Cobalt metal nanoparticles have been reported to induce cytotoxicity and oxidative stress to a greater extent than free cobalt ions [6,8,9,29]. In these studies, the associated cobalt particle toxicity was suspected to be due to the synergistic effect of cobalt released into the culture medium and/or after particle solubilization within the cells. The poorly soluble Co_3_O_4_ nanoparticles have been shown to be less toxic than free cobalt ions [10,13], but caused rapid induction of ROS, with ROS levels higher than those induced by cobalt ions [10,11]. From these studies, it was suggested that the weak amount of extracellular solubilized cobalt in the culture medium could not be responsible for the observed toxicity, while toxicity due to the intracellular solubilization of Co_3_O_4_ nanoparticles following their internalization could not be excluded. However, to date no study has been reported that addresses the intracellular solubilization of cobalt from the Co_3_O_4_ particles, and the relationship with the toxicological effects. The use of high-sensitivity analytical techniques allowed us to demonstrate the relationship between the overall toxicity and the intracellular solubilization of cobalt from low-solubility cobalt oxide particles (Figure 9).

Our results on BEAS-2B cells, a non tumorigenic cellular model of airway epithelium, indicate that human bronchial epithelial cells exposed to Co_3_O_4_ microparticles are in fine exposed to a low concentration of solubilized Co, and may be prone to health effects due to long term exposures to soluble Co. As soluble cobalt compounds such as CoCl_2_ are genotoxic and are classified as potential carcinogenic agents (classified by IARC as Group 2B), our results indicate that solubilized cobalt from Co_3_O_4_ particles could exert a genotoxic and carcinogenic effect. Genotoxic effects can be observed at very low concentrations without a threshold. In order to further elucidate cobalt oxide toxicity mechanisms, our next purpose will be to perform a comprehensive genotoxicity study (DNA breaks, chromosomal damage).

## Conclusion

In conclusion, our study reveals for the first time that the overall toxicity of low-solubility metal oxide compounds such as Co_3_O_4_ cobalt oxide particles is due mainly, and perhaps totally, to their intracellular solubilization. This finding was made possible by the implementation of accurate and sensitive analytical techniques, based on single-cell chemical imaging and ICP-MS analysis of the solubilized cobalt fraction released from particles after cell lysis. Both techniques produced convergent results and highlighted the considerable contribution of analytical techniques to the understanding of complex mechanisms of toxicity of particulate compounds. We also showed that cobalt oxide particles were rapidly taken up by BEAS-2B cells, clathrin-mediated endocytosis probably being the main pathway for internalization of these particles. Cobalt oxide particles were located within the lysosome, where the acidic pH and hydrolytic enzymes enhance their solubilization. Our results, and studies from other research groups [7,15,16,31], confirm the importance of intracellular particle solubilization-associated mechanisms of toxicity. Our results apply to the BEAS-2B cell line, they should now be confirmed with other lung cell models to take into account the ‘cell vision effect’ [50]. In addition, cobalt oxide particles are cleared slowly from the lung and can remain in pulmonary cells for several months or years [3,18-21], thus representing a source of internal solubilized cobalt exposure. We have also shown that cobalt oxide particles would produce significant toxic effects only at very high concentrations, which are not relevant to environmental or even accidental occupational exposures.

## Methods

### Reagents

LHC9, LHC basal medium and trypsin were purchased from Life Technologies. Co_3_O_4_ particles were supplied by Merck. The manufacturer’s quality control sheet indicated a purity of 98.4%. Co_3_O_4_ stock solutions (10 mg.mL^−1^ cobalt in MilliQ H_2_O) were sonicated for 15 min with an Autotune sonicator (Fisher Scientific) operated at 750 W, and then stored at −20°C [26]. CoCl_2_.6H_2_O was purchased from Sigma and prepared similarly without the sonication step. Stock solutions were diluted in culture medium and sonicated for 15 min (Co_3_O_4_) or 1 min (LB-3) before further dilution and addition to cell culture plates. Nitric and hydrochloric acid of high purity grade (HNO_3_ 65% Normapur and HCl 35% Rectapur) were purchased from VWR Prolabo (France) and PBS from Gibco BRL. Cobalt solutions for the ICP AES/MS calibration curves were prepared from standard solutions (1000 μg.mL^−1^), which were purchased from SPEX (SPEX Certiprep Company). Negative controls were performed with Latex Beads (LB-3, Sigma) of size close to those of the Co_3_O_4_ particles. RIPA lysis buffer was purchased from Santa Cruz and Mini EDTA-free protease inhibitor cocktail from Roche. The CellTiter-Glo®Luminescence Cell Viability Assay was purchased from Promega. PALL Acrodisc filters (0.1 μm) were purchased from VWR. Other reagents were purchased from Sigma.

### Cobalt oxide particle characterization

Particle size distribution was assessed by dynamic light scattering (DLS) measurements of particle suspensions using a Nano ZS ZetaSizer (Malvern). Each suspension was vigorously shaken prior to analysis. We assessed the effects of a more drastic physical dispersion method on particle size distribution. For this, the samples were sonicated with an Autotune 750 W ultrasonicator (Fisher Scientific) with the microprobe set at 40% power. Sonication was performed on melting ice to avoid sample heating. Particle suspensions were then poured into clean disposable cuvettes. At least three independent DLS runs were performed at 25°C, with each consisting of the average of fifteen consecutive measurements.

### Cell cultures and exposure to cobalt oxide particles

The BEAS-2B human bronchial epithelial cell line was obtained from the American Type Culture Collection (CRL#9609). BEAS-2B cells are transformed human bronchial epithelial cells. They have been extensively used for particle toxicity studies, including the assessment of cellular uptake routes and the study of cellular stress response [32,36].

Cells were cultured in LHC9 medium in flasks or in precoated plates. The coating was performed using a mixture of BSA (0.01 mg.mL^−1^), human fibronectin (0.01 mg.mL^−1^) and collagen (0.03 mg.mL^−1^) prepared in LHC basal medium. All cell cultures were maintained in 75 cm^2^ cell culture flasks at 37°C in 5% CO_2_ at 95% humidity. The cells were passaged by trypsinization (0.25% trypsin/2.6 mM EDTA) at 70-80% confluency every 3–4 days.

Normal primary human bronchial epithelial cells (NHBE) were obtained from Clonetics™ (Lonza, Switzerland). Cells were maintained in bronchial epithelial basal medium (BEBM) (Lonza, Walkersville, MD) supplemented with growth factors from the SingleQuots kit (as recommended by the manufacturer; Lonza, Walkersville, MD) to make BEGM, which contains a retinoic acid (RA) supplement. All cell cultures were maintained in 75 cm^2^ cell culture flasks at 37°C in 5% CO_2_ at 95% humidity. The culture medium was changed every 48 h until the cells reached 70 − 80% confluency; cells were then passaged by trypsinization (trypsin neutralizing solution).

For Co_3_O_4_ exposure experiments, cells were seeded at 10,000 cells/cm^2^. The LHC9 medium was replaced 24 h later with cobalt-containing medium and cells were incubated for 72 h, except for the clonogenic assay. Exposure solutions (0–1000 μg.mL^−1^ Co) were freshly prepared by diluting the appropriate volume of stock solution in culture medium, and sonicated for 15 min as described above, prior to addition to cell culture plates. As exposure was performed in 6- or 96-well plates, or in 175 cm^2^ flasks, the volume of exposure solution was strictly adjusted to the surface of the well or flask. This precaution is mandatory to obtain a perfect correspondence between concentrations expressed either in μg.mL^−1^ or in μg/cm^2^. A concentration of 100 μg.mL^−1^ corresponded to 33 μg/cm^2^.

### Cell toxicity assays

The effects of Co_3_O_4_ particles, CoCl_2_, and latex beads (LB-3) on the viability of human BEAS-2B cells were evaluated using two methods: the CellTiter-Glo assay and the Clonogenic assay.

First, cell viability was measured using the Promega CellTiter-Glo™ Luminescence Cell Viability Assay. This test allows the measurement of the amount of intracellular ATP, this metabolite being directly linked to the number of metabolically active cells. For this, cells were plated at 10,000 cells/cm^2^ in white 96-well plates and grown for 24 h. The medium was replaced with Co_3_O_4_, CoCl_2_, or LB-3 solutions and the plates were incubated for a further 72 h period. The protocol described by the manufacturer was modified to avoid particle interference during luminescence reading [26]. The Promega CellTiter-Glo reagent (100 μL) was added, and after 20 min incubation plates were centrifuged (900 g, 5 min) to pellet the Co_3_O_4_ particles. 100 μL supernatant from each well was transferred into an empty plate. These conditions for Co_3_O_4_ particle removal were shown to allow full recovery of the signal. The luminescence was measured on a plate reader (LumiStar, BMG). For each condition, a minimum of three independent assays were carried out, each being performed in quadruplicate. In order to have the highest signal, the gain was adjusted to 90% of the maximum read signal, and a 5 s integration reading was used. Variability among experiments was taken into account, and all values were expressed in% (mean luminescence for a sample divided by the mean luminescence for unexposed cells X 100). Data were analyzed using the Prism 4 v4.0 software (GraphPad software). The half-maximal inhibitory concentration (IC50), was calculated as the half-maximal (50%) inhibitory concentration (IC) of cobalt. The IC25 and IC75 were also determined.

We also performed the clonogenic assay described by Puck and Markus [51] and Franken *et al.*[52]. Exponentially growing cells (BEAS-2B and NHBE) were harvested and seeded in 6-well microplates (Nunc, Denmark) at a density of 200 cells/well in 3 mL cell culture medium. Cells were allowed to attach for approximately 16 h, a time shorter than the population-doubling time of the cell line, which is reported to be around 24 h. The medium was then replaced with Co_3_O_4_ (0–1000 μg.mL^−1^ Co) or LB-3 (0–1000 μg.mL^−1^) solutions and the plates were incubated for a further 24 h period. After 24 h exposure, the medium was replaced with fresh culture medium. This medium renewal was performed twice per week. BEAS-2B and NHBE cells were cultured over 10 days corresponding to the time needed to obtain colonies, a colony being defined as at least 50 clones of one initial cell. The colonies were stained with crystal violet (0.5%) for 30 min and rinsed with water. Viable colonies containing more than 50 cells were counted manually. The results were normalized with the unexposed control and expressed as colony forming efficiency, which is the ratio of the mean number of colonies in the treated condition over the mean number of colonies in the control condition. Data were analyzed using the Prism 4 v4.0 software (GraphPad software). The half-maximal inhibitory concentration (IC50) was calculated as the half-maximal (50%) inhibitory concentration (IC) of cobalt.

### Solubilization ratio measurement of Co_3_O_4_ particles in culture media

Elemental analyses were performed with an Activa ICP-AES spectrometer (Jobin Yvon) or an XSeries^II^ICP-MS (ThermoElectron). For ICP-AES analyses, an external standard calibration curve using three wavelength emission lines at 228.616, 237.862, and 238.892 nm, with 5 s integration time, was used. For each wavelength, data were the mean value of five replicates and the final concentration was the mean value obtained for the three wavelengths. For ICP-MS analyses, an external standard calibration curve was used by monitoring ^59^Co isotope combined with Indium internal calibration; data were the mean value of ten replicates.

The reference medium for BEAS-2B cells used in this study is LHC9 (pH 7.4), which is widely used for the culture of primary human lung cells. This medium comprises basal medium (LHCB), which mainly contains salts and amino acids supplemented with proteins and growth factors (retinoic acid, insulin, epidermal growth factor, bovine pituitary extract, hydrocortisone, and triiodothyronin) but does not contain fetal calf serum.

Three independent sets of Co_3_O_4_ particle suspensions (50, 170, and 600 μg.mL^−1^ cobalt) were prepared in the appropriate volume of LHC9 and were equilibrated respectively for 3 and 7 days at 37°C. The total cobalt concentration in each sample was measured by ICP-AES or ICP-MS. The samples were evaporated to dryness and digested consecutively in ultrapure HCl and HNO_3_, which were also evaporated to dryness. The residue was further resolubilized in 1 mol. L^−1^ HNO_3_ for elemental analysis. The solubilized fraction of cobalt from the Co_3_O_4_ particles was also measured from three independent sets. The particle suspensions (50, 170, and 600 μg.mL^−1^ cobalt) were equilibrated for 3 and 7 days at 37°C, and further centrifuged for 1 h at 160,000 g in order to separate the cobalt particles from the solubilized cobalt fraction kept in the supernatants, which were filtered through a 0.1 μm membrane filter. The absence of particles in the supernatant was verified by DLS. The supernatants were further treated as described above and the cobalt concentration was measured by ICP-MS in each of them. The ratio of solubilized cobalt fraction over the total cobalt content, expressed in percentage, allowed reaching the dissolution ratio of the cobalt oxide particles. Similar measurements were performed in ALF medium (pH 4.5), prepared according to the method of Marques et al. [53]. Data are reported as the means with the relative combined uncertainty, taking into account the uncertainty associated with the total cobalt concentration (3% at k = 2) and the Co concentration in the supernatants (3% and 7% at k = 2 for concentrations > 0.05 μg.mL^−1^ and < 0.05 μg.mL^−1^, respectively).

### Cobalt oxide particle uptake by BEAS-2B cells

Cellular uptake of particles can be directly assessed by measuring the increase in scattering (side scatter or SSC) of the laser light in a flow cytometer [54,55]. It has previously been shown that cell fluorescence after phagocytosis of fluorescent latex beads increases in the same manner as the mean SSC of the cells [55]. BEAS-2B cells were treated with cobalt particles at 200 μg.mL^−1^ Co during different periods (0.5 to 5 h). After specific treatment times, the cells were trypsinized, centrifuged, and washed in PBS. Cell suspensions were analyzed using a FACSCalibur flow cytometer (Beckton Dickinson) with the following parameters: excitation at λ_ex_ 488 nm and detection at λ_em_ 530 ± 15 nm. Right-angle light scatter or side scatter (SSC) was measured through a BP 488 nm filter positioned at 90° incident to the cell flow, while fluorescence emission from latex particles was recorded with a BP 520 nm filter. Particle uptake was monitored using the Stringer et al. method [54]. Because ingested or bound particles induce a more granular morphology of the epithelial cells, the laser light is scattered to a greater extent by these cells. Thus, the SSC signal is directly correlated to the quantity of particles bound or ingested. Particle uptake was monitored with the increased SSC signal from a univariate histogram of SSC versus the number of events (after selection of viable cells by gating from a bivariate histogram of forward-angle light scatter versus SSC). As unbound particles were substantially smaller than epithelial cells, they were removed from the forward-angle light scatter versus SSC window by adjusting the electronic threshold settings. Series of 10,000 events were counted for each condition. Data were processed using Beckton Dickinson CellQuest software.

BEAS-2B cells were treated with either 5 μg.mL^−1^ cytochalasin B, 50 μM amiloride, 0.5 or 5 mM methyl-β-cyclodextrin, or 2, 10 or 20 μg.mL^−1^ chlorpromazine. Cells were preincubated in LHC9 medium containing these drugs at a 2X concentration for 1 h at 37°C and 5% CO_2_. Cobalt oxide particles were then added to the cells in an equal volume of medium (200 μg.mL^−1^ Co), and further incubated for 5 h at 37°C and 5% CO_2_. After exposure to particles and inhibitors, the cells were washed with PBS, trypsinized, and processed for flow cytometry as described above. Drug concentrations were chosen according to previous work [56,57].

### Transmission electron microscopy (TEM)

BEAS-2B cells were exposed to Co_3_O_4_ particle suspensions (1, 5, and 20 μg.mL^−1^ Co) for 1 h and 24 h. These low concentrations were chosen to allow the cutting of ultrathin sections, as cobalt oxide is a very hard material which damages the cutting device as well as the cell layer. After exposure, the medium was removed and the cell layer was washed with PBS. Cells were then fixed with 2.5% glutaraldehyde in 0.1 M sodium cacodylate buffer (pH 7.4) for 4 h at room temperature. After fixation, cells were washed three times for 5 min in the sodium cacodylate buffer. Samples were post-fixed with 1% osmium tetroxide in same buffer for 1 h, dehydrated through a graded ethanol series, and finally embedded in Epon 812. All chemicals used for histological preparation were purchased from Electron Microscopy Sciences (Hatfield, USA). Ultrathin 120 nm sections were obtained using an UCT ultramicrotome (Leica, Microsystems GmbH, Wetzlar, Germany) mounted on copper grids, and examined in a Tecnai 12 G^2^ Biotwin scanning transmission electron microscope (FEI Company, Eindhoven, the Netherlands) coupled with a Megaview III CCD camera (Olympus Soft imaging Solutions GmbH, Münster, Germany). At least 50 photographs of detailed local structures of cells were taken, analyzed and compared for each condition. Several cobalt particles were detected and focused with a Phoenix energy dispersive X-ray microanalyser (EDAX Inc., Mahwah, USA) equipped with a Sapphire Super Ultra Thin window detector.

### Confocal microscopy

BEAS-2B cells were grown on glass coverslips. They were incubated with Co_3_O_4_ particles (20 μg.mL^−1^ Co) for 24 h at 37°C. After three washes in phosphate buffered saline (PBS), cells were fixed for 15 min with 4% paraformaldehyde and neutralized with 10 mM NH_4_Cl_2_ in PBS for 15 min. After three washes in PBS, cells were permeabilized with PBS containing 0.1% Triton X-100 and 0.5% bovine serum albumin. To detect endogenous proteins, coverslips were incubated with the following primary antibodies: monoclonal mouse anti-EEA1 for early endosomes (BD Biosciences, Palo Alto, CA, USA; used at 1:50) and monoclonal mouse anti-GM130 for golgi apparatus (BD Biosciences, Palo Alto, CA, USA; used at 1:200). After three washes with PBS, the coverslips were incubated with FluoProbes®594 fluorochrome-coupled secondary anti-species antibodies (Interchim, Montluçon, France; used at 1:400). For lysosome staining, cells were incubated before fixation with 100 nM LysoTracker Red DND-99 (Molecular Probes) at 37°C for 1 h. Cells were washed and the coverslips were mounted on slides using Mowiol 4–88 as antifading agent in a glycerol-based mounting medium. Confocal imaging was performed using a Leica TCS SP2 confocal microscope with a 63X oil immersion objective, by sequential excitation at 488 nm and 543 nm, at the Bordeaux Imaging Center Ibisa platform (http://www.bic.u-bordeaux2.fr). Co_3_O_4_ particles were detected by reflection mode at 488 nm. Adobe Photoshop software (Adobe Systems, San Jose, CA) was used for image processing.

### ICP-MS analysis of intracellular solubilized cobalt and zinc content

Elemental analyses of cobalt and zinc in BEAS-2B cell lysates were performed by ICP-MS after cells exposure to several Co_3_O_4_ particle suspensions for 72 h. After exposure of the cells to 0, IC25 and IC50 respectively (0, 50 and 170 μg.mL^−1^ of cobalt) for 72 h, cells contained in the flasks were harvested, washed, counted using the trypan blue exclusion method on an automated cell counting system Cedex (Roche Innovatis), and further submitted to lysis. Hence for each experiment, the cobalt content was measured within a known number of cells, rendering possible the normalization. Data were collected from two independent experiments, each performed in duplicate. LHC9 alone was used for the control. Cells were plated at 10,000 cells/cm^2^ in 175 cm^2^ precoated flasks and were allowed to attach for approximately 16 h. The medium was replaced with suspensions of Co_3_O_4_ particles prepared in LHC9 medium, corresponding to 0, IC25 and IC50 (0, 50 and 170 μg.mL^−1^) cobalt, respectively. After incubation for a further 72 h period, the medium was removed, the cells were carefully rinsed with PBS and detached with trypsin. The cells were lysed with 2 mL RIPA lysis buffer containing protease inhibitor cocktail tablet (one tablet for 10 mL lysis buffer). The cellular extracts were sonicated five times for 5 s, incubated on ice for 30 min, and centrifuged at 160,000 g for 60 min at 4°C. The supernatants were carefully filtered through a 0.1 μm membrane filter for further analysis. Supernatants were successively digested with ultrapure HCl and ultrapure HNO_3_. Acids were evaporated to dryness and the residue digested again in HNO_3_/HCl mixture, in which it was further analyzed. Intracellular solubilized cobalt and zinc content were expressed as fg/cell. In each case, data were the mean values of four experiments, with relative combined uncertainties taking into account the uncertainties related to the standards preparation, the concentration measurement in exposure solutions (3% for cobalt at k = 2 and 10% for zinc at k = 2), the intracellular amount determined by ICP-MS (10% at k = 2) and the cell count (25% at k = 2). For all experiments, cobalt and zinc concentrations were measured experimentally in the exposure solutions and the zinc concentration was found to be in agreement with the expected concentration in LHC9 [58].

### PIXE microanalysis

Cells were grown on polycarbonate foils and exposed for 24 h to Co_3_O_4_ particles at cobalt concentrations of 0, 50 and 170 μg.mL^−1^ (CTRL, IC25, IC50). Cells were cryofixed and freeze-dried to preserve both the chemical and morphological distributions, according to established protocols [59]. Ion-beam quantitative imaging using PIXE and RBS (Rutherford Backscattering Spectrometry) were performed simultaneously with a proton beam of 3.0 MeV energy on the nano-beamline of the AIFIRA facility (Applications Interdisciplinaires des Faisceaux d’Ions en Région Aquitaine) at CENBG (Centre d’Etudes Nucléaires de Bordeaux Gradignan). The proton beam is provided by a 3.5 MV single stage electrostatic accelerator designed by the High Voltage Engineering Europa B.V. company (Singletron). PIXE is a well established analytical technique for the quantitative determination of the elements in various types of materials, including biological samples [60]. The detailed protocols for accurate quantitative element analysis in biological samples used in our study have been described by Carmona et al. [59] and are based on previous works using the combination of PIXE and RBS analyses [61]. RBS is performed in complement to PIXE to determine the matrix composition, C, N, and O in the case of biological samples. PIXE analysis enables the detection of trace elements of Z > 11 with a detection limit of about 1 to 10 μg/g, whereas RBS analysis is carried out to determine the sample mass and incoming charge. The combination of both methods results in fully quantitative analysis: trace element content normalized by the local mass of the sample [59]. The proton beam was focused down to a 0.8 μm spot size, resulting in a 350 pA beam current on target, for the analysis of single cells to determine *in situ* the trace element content (Mg, P, S, K, Ca, Fe, Co and Zn) in subcellular compartments (nucleus, cytoplasm). The square scanning side size typically ranged from 20 to 50 μm with 128×128 pixel resolution. X-ray analyses were performed in a two-Si(Li) detector configuration (e2v Scientific Instruments, Sirius 80 mm^2^/Be/PIXE (CENBG custom) and RBS with a Si detector (PIPS, PD series, 25 mm^2^, Canberra). RBS and PIXE raw data were converted with a Labview data converter routine (CENBG homemade) and analyzed with SIMNRA [62] and GUPIXWIN [63] softwares.

## Competing interests

The authors declare that they have no competing interests.

## Authors’ contributions

RO, CB and VM participated in the experiments and drafted the manuscript. CD, VA, MF, CG, SR, LP and MJ carried out the experiments, analyzed the data and revised the manuscript. VM, RO, CB, AC designed the study, analyzed the data, and revised the manuscript. All authors read and approved the final manuscript.
